# The social imaginary in the daily lives of family caregivers during the prevention of childhood tuberculosis in southern Lima, Peru

**DOI:** 10.17843/rpmesp.2025.423.14430

**Published:** 2025-09-29

**Authors:** Jhon Alex Zeladita-Huaman, Rosane Gonçalves Nitschke, María Josefa Arcaya-Moncada, Gladys Carmela Santos-Falcón, Roberto Zegarra-Chapoñan, Maria Lígia dos Reis Bellaguarda

**Affiliations:** 1 Universidad Nacional Mayor de San Marcos, Lima, Peru. Universidad Nacional Mayor de San Marcos Universidad Nacional Mayor de San Marcos Lima Peru; 2 Universidad Federal de Santa Catarina, Florianópolis, Brasil. Universidade Federal de Santa Catarina Universidad Federal de Santa Catarina Florianópolis Brazil

**Keywords:** Tuberculosis, Caregivers, Activities of Daily Living, Chemoprophylaxis, Qualitative Research, Family Nurse Practitioners

## Abstract

**Objective.:**

To interpret the social imaginary of family caregivers regarding the daily practices of preventive tuberculosis therapy in children.

**Materials and methods.:**

A qualitative, descriptive-interpretive study, from the perspective of comprehensive and everyday sociology. Fifteen caregivers of children who started preventive tuberculosis therapy in four health facilities in South Lima were interviewed between December 2020 and May 2021. The in-depth interview was used as a technique, and a virtual interview guide as the instrument. The analysis was conducted using the thematic analysis technique.

**Results.:**

Three themes emerged: identifying strengths and limits in coping with tuberculosis; the impact of the social imaginary on sensitive care practices during preventive treatment; and interpreting the social construction of TB and its preventive practices.

**Conclusions.:**

Strengths and limits were evidenced in the daily lives of caregivers while coping with and preventing tuberculosis. Likewise, in their sociability, they provided sensitive care based on their experiences, beliefs, feelings, and social bonds; they showed relative sensitivity and creativity in the face of the imposition of clinical rationalism. Furthermore, it was found that their ordinary knowledge and understanding of the disease were mediated by fears, uncertainties, and beliefs. Finally, their capacity to interpret and give meaning to the lived experience of the other is highlighted.

## INTRODUCTION

Tuberculosis (TB) is a serious threat to public health due to its impact on the individual, family, and society ^1^. In children and adolescents, the percentage of cases has increased globally, from 8% of the total reported in 2018 ^2^ to 12% in 2023 ^3^. On the other hand, although the prevalence of TB in household contacts is relatively low ^4^, minors who have been in contact with adults diagnosed with TB have a higher risk of infection ^5^.

In this context, Peru, in accordance with the recommendations of the World Health Organization ^6^, implemented TB preventive therapy (TPT), which consists of the daily administration of isoniazid for six months under the supervision of a family caregiver at home. However, only 63.2% of registered contacts under five years of age started this treatment in 2024 ^7^.

Among the caregiver-related factors associated with non-compliance with TPT, food insecurity ^8^, level of knowledge and perceptions ^9^, stigma, and poor coordination with health personnel ^10^ have been described. In addition, it is recognized that social representations of TB are constructed and reinforced not only from knowledge but also from life experiences ^11^. These elements of daily life, imbued with emotions and affections, influence the caregiver’s perception and commitment, affecting their decision-making and, consequently, adherence to TPT. Therefore, there is a need for qualitative studies that explore the daily life of the caregiver, understood as “the way of living of human beings that is shown in the day-to-day, through their interactions, beliefs, values, meanings, culture, symbols, which shape their process of living, in a movement of being healthy and sick, directing their life cycle” ^12^.

On the other hand, the social imaginary, understood as the set of beliefs, representations, and values that give meaning to daily practices, can be approached from Maffesoli’s proposal to understand its collective character ^13^. In caregivers, this construct also shapes the care experience, linking the fear of disease contagion with hope and the sense of symbolic protection that comes from the daily ritual of supervising and administering preventive treatment ^14^. The collective imaginary influences the daily lives of these caregivers when they create perceptions and express feelings about pediatric TB; thus, it conditions the acceptance of the disease, the interpretation of symptoms, care practices, the organization of routine, and adherence to treatment.

Qualitative studies that address the daily lives of people affected by TB indicate that negative feelings and discrimination affect adherence to treatment and their daily activities ^15^^,^^16^. However, no research has been found that delves into subjective experiences, nor that examines how the families of children on TPT perceive, interpret, and face TB in their daily lives. Therefore, the objective of this study was to interpret the social imaginary of family caregivers regarding the daily practices related to TPT in children. The information will allow for the reorientation of the practice of health personnel to promote adherence to TPT and to implement comprehensive education and counseling strategies in the prevention of childhood TB.

**KEY MESSAGES**

Motivation for the study. Children in contact with adults diagnosed with tuberculosis are at higher risk of infection, and there are few qualitative studies on the daily lives of families when a member is affected by tuberculosis.

Main findings. Family caregivers express limits and strengths in coping with the disease and provide sensitive care to the child in their daily lives, considering their social imaginary.

Implications for public health. Considering the social imaginary reflected in the daily life of family caregivers is fundamental to promoting adherence to preventive tuberculosis therapy and contributing to the prevention of the disease.

## MATERIALS AND METHODS

### Design

A qualitative, descriptive-interpretive approach was used, aimed at generating knowledge and understanding specific situations. This design allows for the analysis and revelation of meanings rooted in people’s experiences ^17^. The study followed the recommendations of the COREQ instrument ^18^ (supplementary material 1) to ensure transparency and quality. The research was developed within the framework of a doctoral thesis in Nursing ^19^, from which the data and analysis for this article were extracted.

The theoretical-methodological reference used was based on the comprehensive and everyday sociology proposed by Maffesoli. This perspective constitutes a solid framework because it values sensitive reason, that is, it recognizes the importance of emotions, symbols, and shared experiences in daily life ^13^. From this perspective, it explores how caregivers construct a social imaginary around TB and its prevention. Likewise, through the notion of neo-tribalism, it is understood how caregivers form support communities, share experiences, and generate a collective identity. For its part, Maffesoli’s aesthetics and ethics of the everyday facilitate the analysis of how the disease is lived and faced from a more human and less rationalist perspective, revealing the symbolic and emotional dimension of the caregiver’s experience ^20^.

Maffesoli proposes five theoretical premises of sensitivity, defending sensitive reason ^21^. The first: “critique of dualism,” postulates that all thought or action is interconnected and mutually influenced by two opposing and complementary forces or attitudes, which are precisely indefinable because they reflect various potentialities ^22^, that intersect in a complex dialectic reflecting the dynamics of social life and the diversity of human experience, such as reason and imagination. Maffesoli presents the notion of strength as the capacity or internal force of liberation of people ^23^, to create shared meanings, build solidarity networks, and maintain forms of “sociability” based on proximity and affectivity ^24^. In addition, he proposes the notion of limit, which implies determination or commitment, representing a framework of containment for survival in situations a person faces in their daily life, which protects them from adverse events characteristic of their condition ^23^.

The second: “formism,” is a modulation that allows for “describing the contours from within, the limits and the necessity of situations and representations that constitute the everyday” ^21^. The third, “relativistic sensitivity,” holds that there is no single reality, but instead that life experiences are heterogeneous and plural, which requires a broad and comprehensive understanding of social phenomena. The fourth: “stylistic research,” highlights the importance of ensuring reciprocity between form and empathy, without forgetting scientific rigor. The fifth: “libertarian thought,” defends the importance of the “freedom to see” ^21^.

### Participants

The study was conducted in first-level health care facilities located in the Directorate of Integrated Health Networks of South Lima, Peru. Two health centers with 12-hour care that provide basic services without hospitalization, and two maternal-child centers that offer basic, specialized, and hospitalization services.

The study population consisted of family caregivers of the children (mothers, grandmothers, or stepmothers), responsible for the minor’s care. These individuals assumed the responsibility of collecting isoniazid weekly from the health facility and administering it daily to the child at home. Generally, they were patients diagnosed with TB, although in some cases, they were household contacts without the disease.

Caregivers who lived within the jurisdiction of the selected health facilities and were over 18 years of age were included; those with foreign nationality were excluded. The final number of participants was 15 caregivers; with them, data saturation was reached, and they were selected through convenience sampling. Two caregivers declined to participate in the study because they did not have time available for the interviews.

### Procedures

Data collection was carried out between December 2020 and May 2021, led by the main author, who had no prior relationship with the participants. The researcher held a master’s degree, was pursuing a doctorate in Nursing, and had experience in qualitative research. Initially, the project was presented to the head of each health facility and then to the health personnel. The identification of caregivers was done in coordination with said personnel and was corroborated with the index case (person affected by TB). Ten caregivers were contacted at the health facility, and in five cases, through a home visit.

Due to the restrictions established during the COVID-19 pandemic, once a potential participant was identified, they were invited to join the study through the informed consent process. If they accepted, an interview date was coordinated. The virtualized in-depth interview technique was used ^25^, in a single session lasting an average of 45 to 60 minutes. Arrangements were made by phone; when the participant had a device with an internet connection and agreed to a virtual interview, it was conducted via platforms like Zoom or Google Meet. All sessions were recorded, and the caregivers were in their homes during the interview.

An in-depth interview guide was used (supplementary material 2), which began with a guiding question to explore the experiences of the caregivers of children on TPT. From this, specific follow-up questions were formulated about how psycho-emotional, cultural, social, and economic aspects influenced the perception of TB, the implementation of preventive practices, and adaptation strategies in their daily lives. Before data collection, a pilot of the interview guide was conducted in one of the selected health facilities. This procedure allowed for adjusting the wording of the questions, ensuring their clarity, estimating the duration, and evaluating the flow of the interview.

### Data recording, organization, and analysis

All interviews were recorded in digital format and later transcribed, generating a text document. The analysis was carried out using thematic content analysis ^26^, which includes stages of pre-analysis, material exploration, and treatment of results. The data obtained from the interviews were transcribed, followed by a fluctuating reading of them; then, they were organized, forming the units of analysis, to establish codes, which later constituted themes through open coding.

The initial coding of the data was done by the principal investigator and reviewed by a second investigator with experience in qualitative research. This review allowed for adjusting codes and improving the coherence and rigor of the analysis. In addition, to ensure methodological rigor, the quality criteria proposed by Salgado Lévano ^27^ were applied. Credibility was ensured by validating the findings with the participants. The context of the study is described to facilitate the transferability of the results. Dependability was achieved through consistent immersion in the field, and confirmability was ensured through the review of the results by expert researchers in the methodology and theoretical framework used in this study.

The ATLAS.ti version 8.0 software was used for data analysis. The coding was done inductively, building an initial codebook from the first five interviews, which generated 50 codes. Subsequently, a validation process was carried out with the subsequent five interviews, with the guidance of an expert in the theoretical framework. Codes were merged and definitions were adjusted to achieve greater coherence. The final result was a consolidated codebook with 36 codes, organized into 10 registration units and three themes for the final analysis.

### Ethical aspects

The project was approved by the Ethics and Research Committee of the Faculty of Medicine of the Universidad Nacional Mayor de San Marcos and the Ethics Committee of the Directorate of Health Networks of South Lima. It was also registered in PRISA (Registry of Health Research Projects). Initially, caregivers were provided with detailed information about the objectives and procedures of the research. Before each interview, verbal informed consent was obtained, and authorization to record the session was requested. The anonymity of the participants was guaranteed by assigning pseudonyms inspired by names from Greek mythology.

## RESULTS

Fifteen caregivers participated, whose characteristics are presented in Table 1.

**Table 1:** Characteristics of the interviewed caregivers.

Pseudonym	Diagnosed with TB	Age	Marital status	Educational level	Relationship
Zeus	Yes	24	Single mother	Completed secondary education	Mother
Hera	No	32	Married	Completed secondary education	Mother
Poseidon	Yes	35	Cohabiting	Completed secondary education	Mother
Aphrodite	Yes	36	Married	Completed secondary education	Mother
Ares	Yes	25	Married	Completed secondary education	Mother
Athena	No	56	Cohabiting	Completed primary education	Grandmother
Hermes	Yes	48	Married	Completed secondary education	Mother
Apolo	Yes	24	Married	Completed secondary education	Mother
Artemis	Yes	45	Married	Completed secondary education	Mother
Gea	No	34	Married	Incomplete secondary education	Mother
Hephaestus	Yes	43	Married	Completed secondary education	Mother
Cronos	No	35	Married	Completed secondary education	Mother
Demeter	Yes	41	Married	Incomplete secondary education	Mother
Hestia	Yes	27	Cohabiting	Completed secondary education	Stepmother
Dionicio	No	40	Married	Incomplete secondary education	Mother

### Themes reflecting the social imaginary in the caregiver’s daily life

The thematic analysis of the statements allowed for the identification of 449 registration units, which form three themes: a) identifying strengths and limits in coping with TB, b) the impact of the social imaginary on sensitive care practices during preventive treatment, and c) interpreting the social construction of tuberculosis and its preventive practices.

### a) Identifying strengths and limits in coping with TB

Comprised of two sub-themes. The participants’ statements illustrating each of these sub-themes are detailed in Table 2.

**Table 2:** Subthemes and statements reported as enablers and barriers for coping with tuberculosis.

Subtheme	Statements
Enablers	*- I would already be dead, even in the hospital, but thank God, because of these medications, I am continuing with my life and also with my children* (Poseidon). *- I don't know if he will have a reaction; I hope not, that he just stays calm. We have to thank God that everything will be okay with our children* (Hera). *- Thank God that in these times there are treatments; it's not like before, when there was no treatment. The people who got sick most of them died. I have fought, thank God, I got through this, and well, here we are* (Hermes). *- They told me about the illness, about the fever for more than 15 days, but I don't remember anymore, and I don't want to think about that illness, because it's horrible* (Hera). *- But, afterward, little by little, I understood. Sometimes, things happen in life that you just have to overcome. I believe that one must learn to survive with everything that happens, and I have to overcome it* (Gea). *- At first, I couldn't go to my house. My family also didn't know what I had. I felt a bit of shame, and I felt lonely. As the months went by, as the nurse told me, "After 2 months you are no longer contagious," I still went with my mask, I talked with my family, and they helped me, they supported me* (Ares). *- My husband gives me a lot of emotional and moral support; he supports me a lot, he takes me to my check-ups, and he goes to pick up my pills, as much as he can* (Athena). *- They treated me well, they explained that it was spread by sneezing or coughing, that we should be careful. The health staff helped me well, I did understand, well, not at the beginning* (Ares). *- Here at the health post, excellent, they told me: "They have to take these pills, which is for prevention," because of what I was going through, it was protection for them. The nurses are wonderful, good people; right away, they attend to you. With their help, I gained access to the SIS (Comprehensive Health Insurance)* (Demeter).
Barriers	*- It came out of nowhere… the illness hit me hard… I stopped eating. It's a great pain, and it's worse to be sick and have your family see you like that; it's sadder. I felt bad because I couldn't do anything, I couldn't work or take care of my daughters, I was sick, I distanced myself a bit from them…I was scared because I thought I would die from it* (Zeus). *- I thought I was going to die, because my lungs felt strange, I couldn't even breathe very well, like now when I take a deep breath…I thought that even if I took the pill, I was going to die, I'm afraid of relapsing, and this time actually dying and leaving my little children, who are so young* (Poseidon). *- At the very beginning, it was like I couldn't work, I just had to be at home, I couldn't exert myself…I was coughing all the time, but after three months, I started to do my things normally* (Zeus). *- When you get sick, you avoid several things, you are not with your family because they discriminate against you… It's like I was marginalized, something like that. When I touched him/her, he/she would tell me: "Don't touch me, you could infect me" (Zeus).* *- I received poor treatment at the medical center itself, discriminatory treatment from the young lady (cleaning staff), which affected me psychologically, because I was very sensitive* (Dionisio). *- They sent me home, the thing is, I was like that for more than a week, I felt sick, and since COVID-19 came, I didn't want to expose myself anymore… I went to a private doctor (Artemis). Because of the virus illness, there is no care in the hospitals, there is nowhere to go. Everything is collapsed, that upsets and worries me* (Athena).

Regarding the sub-theme of strengths, the caregivers’ coping strategies, such as hope and gratitude to God, are interpreted as rituals that give meaning to their experience. The communication of the TB diagnosis to the family, after overcoming fear, functions as an initiation rite that reintegrates them into an affective tribe, providing them with a sense of belonging and support. Health personnel and support networks act as catalysts for the formation of elective communities, which reinforce the true social bond (sociability) and adherence to preventive treatment.

Regarding the sub-theme of limits, the fear of contagion, fear of death, discomfort, and worry reveal social representations of fragility and stigma. The experience of decreased physical strength, which limited the performance of their daily activities, underscores how TB symptoms not only alter biological function but also diminish perceived vital energy, reconfiguring daily life and the individuals’ sense of autonomy. Discriminatory treatment from family members and cleaning staff was seen as an exclusion from the social tribe, breaking bonds of support. Finally, the restrictions due to the pandemic fragmented institutional rituals and support networks, leaving the caregivers in a more vulnerable situation.

### b) The impact of the social imaginary on sensitive care practices during preventive treatment

Three sub-themes emerged. The participants’ statements illustrating each of these sub-themes are detailed in Table 3.

**Table 3:** Subthemes and reported statements on the impact of the social imaginary of sensitive care during preventive treatment

Subtheme	Statements
Adhering to and completing treatment together	*- My son had to take his pill after breakfast, every morning at 8 a.m. ….. We must support them, not leave them alone, always be with them, … Make their food, always be by their side; that is the most important thing, because otherwise they won't get better either* (Gea). *- Now, since I'm here, I'm more attentive to him (son), with his diet so that he has a good breakfast and when he takes his pill, it doesn't sit heavy with him or affect him … Normally, I give him the pills in the morning, after breakfast, mostly with water …* (Athena). *- Children should be taught about the importance of taking medicines. For example, I show my son (14 years old) videos from the internet about the disease or what can happen if he stops taking the pills. Then, I tell him: "If we complete this phase and get through it, great and happy. But, if not, in a year, they will have to give us injections"* (Aphrodite). *- … They would take it after breakfast, I would give it to them in a little juice, since they like strawberry juice, I mixed it with that, and I also added a little 'honey' so they wouldn't taste it …* (Zeus). *- From the moment they told me my son had to take the pills, I gave them to him; although his dad would tell me, "Why are you going to give it to him?" and things like that. However, I gathered my courage and started giving him the pills until he finished his treatment* (Hera). *- Sometimes, I would forget to give them the pills because of their homework, or because of classes. Also, because I had to go to the market, other times because of so many personal worries I have, that was also why* (Poseidon).
Interacting with the child	*- When she started taking it (isoniazid), I told her: "Daughter, this is to prevent, so you don't end up like me, you have to take it." She would grab it, she would tell me: "Mom, give me my medicine"* (Zeus). *- I never told my children, because I didn't want them to find out that I had TB, and they don't ask me much either. I just told them they were little pills they had to take so they wouldn't get anemia or get sick, or anything like that* (Ares). *- He (son) was taking it (preventive treatment), but, like any young person, he kind of didn't want to take it, you have to be after him (son) to get him to take it because he said he was fine* (Athena). *- …she stopped taking it and left it with one month to go, because I think she said she didn't want to take it … She herself told me no, that it made her feel bad, that she lost weight, she practically lost her appetite* (Demeter).
Favorable attitudes toward treatment	*- When they told me my girls were going to start treatment with those pills, I thought that they actually had the illness that I had; that's what I started to think* (Zeus). *- He had to take these pills so he wouldn't get sick from that later on, because he had been in contact with my brother for more than a month, without knowing he had that disease* (TB) (Hera). *- They told me they were not contagious, but that they had the disease, something like that. "… I don't remember very well." They just gave me the pills as a prevention* (Ares). *- The reason I had for my daughters to comply with the treatment was because I didn't want to feel bad, … I didn't want to expose them, maybe not for my health, … because it was a responsibility on my part* (Ares). *- It affected me a lot too, the first month, I almost got depressed because I was saying that it was my fault my daughter was like that, with all those symptoms.* (Dionisio).

Regarding the sub-theme *Being and complying with the treatment together*, the administration of the pills is not just an act of care, but a daily ritual of protection that is influenced by their emotions, beliefs, and social bonds. The caregivers, by applying strategies and fulfilling the task, reinforce a sense of belonging in the “family tribe,” which consolidates their commitment to prevention.

In the sub-theme *Interacting with the child*, caregivers used various communication strategies. One of them was a prevention narrative, which consolidated care practices and facilitated adherence. However, this approach was not uniform. Some caregivers chose to hide the disease, while others had to manage the children’s resistance to the treatment directly.

Regarding the sub-theme *Favorable attitudes towards treatment*, perceptions about the treatment, and the feeling of guilt as a driver of responsibility act as affective mechanisms that solidify the caregiver’s identity, promoting adherence to the ritual and the commitment of the family tribe.

### c) Interpreting the social construction of tuberculosis and its preventive practices

Comprised of three sub-themes. The participants’ statements illustrating each of these sub-themes are detailed in Table 4.

**Table 4:** Subthemes and reported statements on the theme of interpreting the social construction of tuberculosis and its preventive practices.

Subthemes	Statements
Imaginary of practices to prevent TB in the home	*- I also had to separate my plates, my cutlery, all of that to avoid having much contact with my daughters, … the young lady (nurse) also told me: "You can't infect anyone anymore," because I'm taking the medications, I can't infect anyone anymore, but I keep my cutlery and everything separate* (Zeus). *- I put a little mark on my cup, my spoon, on everything. Everyone already knew and didn't grab my cup (Hestia). I worked in a daycare, in the kitchen, we always received talks on nutrition. So, based on that, I have tried to feed my children in one way or another with plenty of legumes, eggs, milk, and vegetables. Although from time to time I would buy them vitamins as a supplement. Also, I started cleaning more. Maybe I exaggerated with the cleaning* (Hermes). *- Tuberculosis is prevented with plenty of food, by eating. That's what they (health staff) told me. I gave him guinea pig meat because I had eaten it when I had anemia before, when I gave birth* (Gea). *- I neglected my diet, I ate on the street. That's when I felt the symptoms. I started coughing up blood. Also, maybe it was when my defenses were low* (Zeus). *- Most people get it from a lack of hygiene, precaution, or low defenses. When your defenses are low, that disease evolves and attacks you. Well, then, you have to eat healthily* (Demeter). *- I had to send him/her to my mother-in-law's house so he/she could be well, so as not to infect them… and sometimes when my daughters come, well since they are little, they want to hug me and I can't. I try to distance myself a little from them because of the disease* (Zeus). *- I told my oldest son that I have a wound in my chest, in my lung, and that I have to wear masks so I can't cough and infect them. I haven't had a cough, but I always wear the mask and try to wash my hands before touching my children's things* (Apolo). *- Nowadays, as we are going through the COVID-19 disease, we don't go out; we stay in the room. He/she always washes his/her little hands with liquid soap* (Hera). *- The nurse told me: "You should live in a ventilated place and sleep separately." But the situation is difficult, no one rents cheaply and there is no money. That's why we broke a small piece of the plywood (wall) to create a little window, allowing air to enter* (Poseidon). *- Also, I open my windows so the sun can come in* (Hera).
Support in the control of contacts	*- When his brother started the (anti-TB) treatment, they evaluated my son and told me he was at risk. So, they did a screening test, they took X-rays of his lungs; they only took a sputum sample from me, but the result came back negative* (Hera). *- They sent my little daughter to get a test. So, I took my daughter to have the test done on her forearm (tuberculin test), and everyone else around my family. Thank God, everyone tested negative* (Artemis). *- It's just that my little son had a bad cough when I started the treatment for TB. But, it seemed like a strong case of bronchitis, they had to take an X-ray, and the pulmonologist, who was the one who told me he had poorly treated bronchitis* (Apolo). *- They took X-rays of his lungs at the beginning. Then, in the middle and at the end…. Afterward, it was the saliva test (sputum smear microscopy), which they also sent me for, constantly like that. Lastly, too, I don't remember, but yes, it was the monitoring they were doing* (Hermes).
Images of TB	*- Well, I know that it's practically a common disease; I've already had relatives who have been through this: 2 brothers, 2 brothers-in-law. So I know it's curable, this disease is contagious if there is no prevention or care…* (Demeter). *- I only knew that TB was a contagious disease and that it affects the lungs. I didn't know much; no one in my family had had it. That's why it was a bit difficult for me to accept the illness* (Ares). *- …, my relatives who supposedly died, it was because they abandoned the treatment because it was harming them, the disease progressed, and they died … if they don't give me pills, I would already be dead, I even ended up in the hospital* (Poseidon). *- It's complicated, it's a very serious disease, because major things can happen, from what they've explained to me .. that it can be cured, but always by following the treatment to the letter. Imagine, major things can happen like death* (Athena). *- They tell me that I had one of three levels, I don't remember very well. I mean, mine was very strong and I wondered what had happened because it had been a long time since I got sick from a cough, from a fever; but this time it was different, it was very strong"* (Poseidon). *- In my brother's case, he was in a serious condition, because he was spitting up blood and he didn't have a cough, neither did I. It was just that one day he went to work, he drank a cold soda, then his back started to hurt, then he started spitting up blood and had a fever* (Demeter). *- Because mine I think is of a lower grade, I think there are different grades of tuberculos*is (Hestia). *- They informed me that it was a vaccine and that with it we would know if we were infected or not. After they gave it to him, four days later, I came with my children, they told me that we were infected* (Poseidon). *- They sent me to give another sputum sample, also to get x-rays … and I saw the doctor. They told me: "Indeed, you have tuberculosis and you must follow the treatment for 6 months"* (Artemis). *- When the nurse calls me, she tells me I have TB number three, I don't remember well, but they told me that meant I could infect my children* (Poseidon).

In the first sub-theme, the *imaginary of practices to prevent TB in their home* is manifested in rituals of hygiene and protection. The separation and marking of utensils, along with “excessive” cleaning, constitute purification rituals that seek to control the fear of contagion and establish symbolic boundaries in the “family tribe.” The practices of social distancing during the COVID-19 pandemic became rites of collective security. The promotion of ventilation, even in precarious conditions, demonstrates the adaptation of this perception to their reality.

The second sub-theme shows how the *symbolic practices of care* extend beyond the immediate family nucleus. Support in contact tracing, by accompanying family members to screening tests, is interpreted as a ritual of solidarity and tribal cohesion. By considering them at risk, the idea of a community of destiny united by a shared threat is reinforced. Facilitating access to health services consolidates this support network as a collective protection mechanism, reinforcing the affective bonds of the “tribe.” 

In the third sub-theme, the *images of TB* are constructed from knowledge and perceptions. The description of TB as a contagious disease reveals social representations based on fear and prevention. The belief that lack of treatment can cause death is a risk narrative that justifies and gives meaning to care practices. The caregivers assign levels of severity to the disease according to the symptoms, which shows that this imaginary is not static but is reconfigured with experience.

## DISCUSSION

This study, aimed at interpreting the social imaginary of a group of caregivers of children on TPT, revealed that when facing TB, they identify strengths and limits in their daily lives. After assuming their role, they offered sensitive care to the child, permeated by emotions, life experiences, beliefs, and social bonds, which manifested in protection rituals and in the construction of a prevention narrative. Simultaneously, they constructed and expressed a particular meaning of TB and its preventive measures, adapting their hygiene practices to their socioeconomic context and ordinary knowledge. These symbolic acts and meanings were shaped by their fears, uncertainties, and beliefs during their experiences with family and health personnel, as well as by a deep sense of responsibility and solidarity.

In the family environment, upon communicating their diagnosis, caregivers with TB received family support (strength), although some faced situations of mistreatment and discrimination (limit). Similarly, qualitative research conducted in Istanbul ^28^ and Colombia ^29^ highlights that social support from family and friends represents the main support for coping with TB. Likewise, another study reported that African Americans in the United States were discriminated against by their relatives ^30^; in addition, it has been documented in Peru that the accompaniment of family and nursing staff allowed patients to overcome discrimination and social stigma ^31^. These supports demonstrate the “entre-ensemble” as it occurs in “being together,” which calls for a dynamic of solidarity, and “feeling together,” which alludes to the ethics of aesthetics ^32^.

Regarding the health personnel of the TB services, the caregivers also had positive experiences, such as guidance and support (strength); however, they reported situations of discrimination (limit), mainly from cleaning staff. Discrimination in TB may be related to the association of the disease with social and cultural stigmas ^33^. The notions of strengths and limitations also emerged in qualitative studies that analyzed the daily lives of nursing students during their training ^22^^)^ and in healthcare for deaf people ^23^.

The sensitive care provided by caregivers is characterized by a duality between reason and feeling, which enables them to deliver holistic and person-centered care that transcends tasks and focuses on the emotional and physical well-being of the person receiving care. This suggests that the way care is offered can impact the experience of the recipient ^22^. In addition, aspects such as empathy and creativity are incorporated to address the psycho-emotional needs, suffering, and anxiety generated by the disease ^34^. Available evidence suggests that maternal sensitive care has multiple important benefits, including promoting attachment security ^35^ and infant brain development ^36^.

By giving meaning to tuberculosis in their daily lives, the participants demonstrated a multifaceted commitment and resilience. A first finding revealed the “ethics of aesthetics,” present in sociability, which enables the rediscovery of the grace of being and feeling together, of connecting with others, and of living healthily and daily ^37^, where empathy and emotional connection with the child were key to treatment adherence. On the other hand, the use of electronic devices and the internet to negotiate treatment compliance suggests that, in postmodernity, there is a synergy between “archaic and technological development” ^32^. This finding is complicated by the fact that, at times, the commitment was a social “mask” that faded in the privacy of the home, due to socioeconomic pressures and the multiplicity of roles that caregivers assume, which led to neglect in the administration of medication. This central contrast reveals the theatricality of daily life and the complexity of the caregivers’ experiences. This alludes to the allegory of the “game of masks,” where people tend to adopt different roles and social masks depending on the context in which they find themselves. In that sense, the “mask” represents the dramatic dimension of the interwoven experience and the theatricality of daily life ^24^.

The analysis of the social imaginary in the daily lives of the caregivers revealed an integration of reason and feeling. The “critique of dualism” ^22^ is reflected in the balance between the rationality to face the TB diagnosis and the reliance on spirituality and faith to find emotional strength. This duality shows that the management of emotions transcends the physical, as it also involves the emotional and spiritual ^21^. On the other hand, the premise of “formism” underscores the priority that caregivers give to harmony in their care practices, seeking an aesthetic of coherence in their actions. “Libertarian thought” emerged in the caregivers’ ability to generate innovative strategies, demonstrating that everyday life is a space of resistance to external imposition. In addition, relativistic sensitivity ^21^ allowed for the understanding that the meaning of TB is not fixed, but rather is based on individual perceptions and the ordinary knowledge ^13^ that caregivers construct from observing symptoms and interacting with health personnel. This knowledge does not oppose scientific knowledge but complements it.

This study has practical implications for TB prevention, considering that knowledge and perceptions are associated with compliance with TPT ^9^. These findings suggest that health personnel should take these aspects into account when implementing educational interventions or providing individualized counseling. Likewise, to achieve a comprehensive approach to the phenomenon of adherence or treatment compliance, it is necessary to unveil the different “masks” that the caregiver wears through accompaniment, cordial treatment, and consideration of their feelings, fears, and anxieties. From a methodological point of view, this research is a pioneer in addressing TB prevention from the sociology of the everyday, describing strengths and limits in coping with the disease; these notions can be identified as internal and external dimensions of the person, in their relationship with family, health personnel, and their environment.

The study was not without limitations. First, being a qualitative study, its findings cannot be generalized to the entire population of caregivers of children on TPT in Peru. Second, due to the restrictions imposed by the COVID-19 pandemic, it was necessary to conduct virtualized in-depth interviews using digital platforms, which, on some occasions, were interrupted by technical problems. Third, regarding communication, crucial non-verbal information was lost, especially during telephone calls, and there were difficulties in establishing adequate rapport with the participants, which, to some extent, affected the richness of the collected data compared to face-to-face interviews. However, an interview guide was available to ensure the standardization of the data collection process, and the researcher in charge had experience in qualitative research. Fourth, the absence of methodological triangulation to ensure the reliability of the data constitutes another important limitation. As a fifth limitation, the context of the pandemic may have influenced the caregivers’ behavior due to increased cohabitation with the children. Therefore, these findings should be interpreted considering the exceptional conditions of that period, which are no longer in effect. Finally, the small number of participants considered until reaching the point of saturation might not fully reflect the diversity of experiences or perspectives on the topic studied; however, this sample was diverse, as it included people diagnosed with TB and contacts without the disease from four health facilities.

In conclusion, this study reveals that the social imaginary in the daily lives of caregivers of children on TPT is a dynamic and complex construct. Strengths and limits were evidenced during the coping with TB in their daily lives. The sensitive care they provided was influenced by their emotions, life experiences, beliefs, and social bonds. Likewise, the knowledge and understanding of the disease are not purely rational but were imbued with the collective imaginary, in which fears, uncertainty, and beliefs function as a filter through which the experience was interpreted and resignified. These findings suggest that, in TB prevention, it is crucial to adopt a more empathetic, spiritual, and personalized approach that values the creativity, empathy, and resilience of the caregivers, considering their experiences and beliefs. Finally, to improve adherence to TPT, it is essential that health personnel be trained in active listening and empathy skills to recognize the “social masks” that hide the fears and problems of the caregivers. In essence, TB prevention must cease to be an imposition and become a process of accompaniment that values inclusion and sensitivity, integrating community support and spirituality.
