# Supplementary material for: The social imaginary in the daily lives of family caregivers during the prevention of childhood tuberculosis in southern Lima, Peru
Source: Rev Peru Med Exp Salud Publica. 2025 Sep 29;42(3):271–80. doi: 10.17843/rpmesp.2025.423.14430 (PMC12679975; doi:10.17843/rpmesp.2025.423.14430)
Supplement: Translation

# Translation: El imaginario social de las cuidadoras familiares en la cotidianidad de la prevención de la tuberculosis infantil en Lima Sur, Perú

**Authors:** Jhon Alex Zeladita-Huaman, Rosane Gonçalves Nitschke, María Josefa Arcaya-Moncada, Gladys Carmela Santos-Falcón, Roberto Zegarra-Chapoñan, Maria Lígia dos Reis Bellaguarda

**Affiliations:** 1 Universidad Nacional Mayor de San Marcos, Lima, Perú.; 2 Universidad Federal de Santa Catarina, Florianópolis, Brasil.

**Keywords:** Tuberculosis, Cuidadores, Actividades Cotidianas, Quimioprofilaxis, Investigación Cualitativa, Enfermería de la Familia

## Abstract

**Objetivo.:**

Interpretar el imaginario social de las cuidadoras familiares sobre las prácticas cotidianas de la terapia preventiva de tuberculosis en niños.

**Materiales y métodos:**

. Estudio cualitativo, de tipo descriptivo-interpretativo, desde la sociología comprensiva y cotidiana. Se entrevistó a 15 cuidadoras de niños que iniciaron terapia preventiva de tuberculosis en cuatro establecimientos de salud de Lima Sur, entre diciembre del 2020 a mayo del 2021. Se empleó la entrevista en profundidad como técnica, y como instrumento, una guía de entrevista virtual. El análisis se realizó mediante la técnica de análisis temático.

**Resultados:**

. Emergieron tres temas: identificando potencias y límites del afrontamiento a tuberculosis; impacto del imaginario social en las practicas del cuidado sensible durante el tratamiento preventivo; interpretando la construcción social de la TB y sus prácticas preventivas.

**Conclusiones:**

. Se evidenciaron límites y potencias en el cotidiano de las cuidadoras durante su afrontamiento a la tuberculosis y su prevención. Asimismo, en su sociabilidad, brindaron cuidado sensible según sus vivencias, experiencias, creencias, sentimientos y vínculos sociales; mostraron sensibilidad relativa y creatividad ante la imposición del racionalismo clínico. Asimismo, se constató que su conocimiento ordinario y la comprensión de la enfermedad estuvieron mediados por miedos, incertidumbres y creencias. Finalmente, se resalta su capacidad de interpretar y otorgar sentido a la experiencia vivida del otro.

## INTRODUCCIÓN

La tuberculosis (TB) constituye una seria amenaza para la salud pública, debido a su impacto en la persona, familia y sociedad ^1^. En niños y adolescentes, el porcentaje de casos se incrementó globalmente, pasando del 8% del total reportado en 2018 ^2^ al 12% en 2023 ^3^. Por otra parte, aunque la prevalencia de TB en los contactos intradomiciliarios es relativamente baja ^4^, los menores que estuvieron en contacto con adultos diagnosticados de TB presentan un mayor riesgo de infección ^5^.

En ese contexto, en el Perú y de acuerdo con las recomendaciones de la Organización Mundial de la Salud ^6^ se implementó la terapia preventiva de TB (TPTB), que consiste en la administración diaria de la isoniacida durante seis meses bajo la supervisión de un familiar cuidador en el hogar. Sin embargo, solo el 63,2% de los contactos censados menores de cinco años iniciaron este tratamiento en el 2024 ^7^.

Entre los factores relacionados con el cuidador que se asocian al incumplimiento de la TPTB se han descrito la inseguridad alimentaria ^8^, el nivel de conocimientos y percepciones ^9^, el estigma y la deficiente coordinación con el personal de salud ^10^. Además, se reconoce que las representaciones sociales sobre la TB se construyen y refuerzan no solo a partir del conocimiento, sino también de las vivencias y experiencias ^11^. Estos elementos de la vida cotidiana, impregnados de emociones y afectos, influyen en la percepción y compromiso del cuidador, afectando su toma de decisiones y, en consecuencia, la adherencia a la TPTB.

Por ello, surge la necesidad de realizar estudios de enfoque cualitativo que permitan explorar la cotidianidad del cuidador, entendida como «la manera de vivir de los seres humanos que se muestra en el día a día, a través de sus interacciones, creencias, valores, significados, cultura, símbolos, que van delineando su proceso de vivir, en un movimiento de ser saludable y enfermo, dirigiendo su ciclo vital» ^12^.

Por otra parte, el imaginario social, entendido como el conjunto de creencias, representaciones y valores que otorgan sentido a las prácticas cotidianas, puede abordarse desde la propuesta de Maffesoli para comprender su carácter colectivo ^13^. En los cuidadores, este constructo también configura la experiencia de cuidado; vinculando el miedo del contagio de la enfermedad con la esperanza y con el sentido de protección simbólica que se desprende del ritual diario de supervisión y administración del tratamiento preventivo ^14^. El imaginario colectivo influye, en el día a día de estos cuidadores cuando crean percepciones y expresan sentimientos frente a la TB pediátrica; de este modo, condiciona la aceptación de la enfermedad, la interpretación de los síntomas, las prácticas de cuidado, la organización de la rutina y la adherencia al tratamiento.

Los estudios cualitativos que abordan la cotidianidad de las personas afectadas por TB señalan que los sentimientos negativos y la discriminación repercuten en la adherencia al tratamiento y en sus actividades diarias ^15^^,^^16^. Sin embargo, no se han encontrado investigaciones que profundicen en las vivencias subjetivas, ni que examinen cómo los familiares de niños en TPTB perciben, interpretan y enfrentan la TB en su vida diaria. Por ello, el objetivo de este estudio fue interpretar el imaginario social de las cuidadoras familiares sobre las prácticas cotidianas vinculadas a la TPTB en niños. La información permitirá reorientar el quehacer del personal de salud para favorecer la adherencia a la TPTB e implementar estrategias integrales de educación y consejería en la prevención de la TB infantil.

**MENSAJE CLAVE**

Motivación para realizar el estudio. Los niños en contacto con adultos diagnosticados de tuberculosis presentan mayor riesgo de infección y existen escasos estudios cualitativos sobre la cotidianidad de las familias cuando un integrante es afectado por tuberculosis.

Principales hallazgos. las cuidadoras familiares manifiestan límites y potencias para el afrontamiento de la enfermedad y brindan un cuidado sensible al niño en su cotidianidad considerando su imaginario social.

Implicancias para la salud pública. Considerar el imaginario social que se muestra en la cotidianidad del familiar cuidador es fundamental para promover la adherencia a la terapia preventiva de tuberculosis y contribuir a la prevención de la enfermedad.

## MATERIALES Y MÉTODOS

### Diseño

Se utilizó el abordaje cualitativo, tipo descriptivo-interpretativo, orientado a generar conocimiento y comprender situaciones específicas. Este diseño permite analizar y revelar significados enraizados en las experiencias de las personas ^17^. El estudio siguió las recomendaciones del instrumento COREQ ^18^^)^ (material suplementario 1) para garantizar la transparencia y calidad. La investigación se desarrolló en el marco de una tesis doctoral en Enfermería ^19^, de la cual se extrajeron los datos y el análisis para la elaboración de este artículo.

El referente teórico-metodológico utilizado se basó en la sociología comprensiva y del cotidiano, propuesto por Maffesoli. Esta perspectiva constituye un marco sólido porque valora la razón sensible, es decir, reconoce la importancia de las emociones, los símbolos y las experiencias compartidas en la vida cotidiana ^13^. Desde esta óptica, se explora cómo los cuidadores construyen un imaginario social en torno a la TB y su prevención. Asimismo, a través de la noción de neotribalismo, se comprende cómo los cuidadores forman comunidades de apoyo, comparten experiencias y generan una identidad colectiva. Por su parte, la estética y ética de lo cotidiano de Maffesoli facilita el análisis de cómo la enfermedad se vive y se enfrenta desde una perspectiva más humana y menos racionalista, revelando la dimensión simbólica y emocional de la experiencia del cuidador ^20^.

Maffesoli plantea cinco presupuestos teóricos de sensibilidad, defendiendo la razón sensible ^21^. El primero: «crítica al dualismo», postula que todo pensamiento o acción está interconectado y se influyen mutuamente por dos fuerzas o actitudes opuestas y complementarias; indefinibles precisamente porque reflejan diversas potencialidades ^22^, que se entrecruzan en una dialéctica compleja que refleja la dinámica de la vida social y la diversidad de la experiencia humana, como la razón e imaginación. Maffesoli nos presenta la noción de potencia como la capacidad o fuerza interna de liberación de las personas ^23^, para crear significados compartidos, construir redes de solidaridad y mantener formas de «socialidad» basadas en proximidad y afectividad ^24^. Además, propone la noción de límite, que implica determinación o compromiso, que representa un marco de contención para la supervivencia ante de situaciones que enfrenta una persona en su cotidianidad, la cual la protege de acontecimientos adversos característicos de su condición ^23^.

El segundo: «formismo», se trata de una modulación, que permite «describir los contornos desde dentro, los límites y la necesidad de situaciones y representaciones que constituyen la cotidianidad» ^21^. El tercero: «sensibilidad relativista», sostiene que no existe una única realidad, sino que la experiencia de vida es heterogénea y plural, lo que exige una comprensión amplia e integral de los fenómenos sociales. El cuarto: «investigación estilística», destaca la importancia de garantizar la reciprocidad entre forma y empatía, sin olvidar el rigor científico. El quinto: «pensamiento libertario», defiende la importancia de la «libertad de mirar» ^21^.

### Participantes

El estudio se realizó en establecimientos de salud de primer nivel de atención ubicados en la Dirección de Redes Integradas de Salud Lima Sur, Perú. Dos centros de salud con atención de 12 horas que brindan servicios básicos sin internamiento, y dos centros materno-infantiles que ofrecen servicios básicos, especializados y hospitalización.

La población del estudio estuvo conformada por familiares cuidadoras de los niños (madres, abuelas o madrastras), responsables del cuidado del menor. Estas personas asumían la responsabilidad de recoger semanalmente la isoniacida en el establecimiento de salud y administrarla diariamente al niño en el hogar. Generalmente, eran pacientes con diagnóstico de TB, aunque en algunos casos, se trataba de contactos intradomiciliarios sin la enfermedad.

Se incluyeron cuidadoras que vivían en la jurisdicción de los establecimientos de salud seleccionados, que tenían más de 18 años; se excluyeron a quienes tenían nacionalidad extranjera. El número final de participantes fue de 15 cuidadoras; con ellas, se alcanzó la saturación de datos y fueron seleccionadas mediante muestreo por conveniencia. Dos cuidadoras rechazaron participar del estudio porque no tenían disponibilidad de tiempo para las entrevistas.

### Procedimientos

La recolección de datos fue realizada entre diciembre del 2020 a mayo del 2021, estuvo a cargo del autor principal quien no tenía vínculo previo con los participantes. El investigador contaba con el grado académico de magister, cursaba el doctorado en Enfermería y poseía experiencia en investigación cualitativa. Inicialmente, se presentó el proyecto al jefe de cada establecimiento de salud y luego al personal de salud. La identificación de las cuidadoras se realizó en coordinación con dicho personal y se corroboró con el caso índice (persona afectada por TB). Diez cuidadoras fueron contactadas en el establecimiento de salud y en cinco casos mediante visita domiciliaria.

Debido a las restricciones establecidas durante la pandemia de la COVID-19, una vez identificada la posible participante se le invitó a integrarse al estudio mediante el proceso de consentimiento informado. En caso de aceptación, se coordinó la fecha de la entrevista. Se empleó como técnica la entrevista virtualizada a profundidad ^25^, en una sola sesión y tuvo una duración promedio de 45 a 60 minutos. Las coordinaciones se realizaron por vía telefónica; cuando la participante disponía de un dispositivo con conexión a Internet y aceptaba la entrevista virtual, esta se llevó a cabo mediante plataformas como Zoom o Google Meet. Todas las sesiones fueron grabadas y las cuidadoras se encontraban en sus domicilios durante la entrevista.

Se empleó una guía de entrevista en profundidad (material suplementario 2) que iniciaba con una pregunta norteadora para explorar las experiencias y vivencias de las cuidadoras de niños en TPTB. A partir de ella se formularon repreguntas específicas sobre cómo los aspectos psicoemocionales, culturales, sociales y económicos influían en la percepción de la TB, la implementación de prácticas preventivas y las estrategias de adaptación en su vida cotidiana.

Antes de la recopilación de datos se realizó un piloto de la guía de entrevista en uno de los establecimientos de salud seleccionados. Este procedimiento permitió ajustar la formulación de las preguntas, asegurar su claridad, estimar la duración y evaluar el flujo de la entrevista.

### Registro, organización y análisis de los datos

Todas las entrevistas fueron grabadas en formato digital y posteriormente transcritas, generando un documento en texto. El análisis se realizó mediante el análisis de contenido temático ^26^, que comprende etapas de preanálisis, exploración del material y tratamiento de resultados. Los datos obtenidos en la entrevista fueron transcritos, haciendo luego una lectura fluctuante de los mismos; luego, se organizaron, conformándose las unidades de análisis, para establecer los códigos, que posteriormente constituyeron temas, mediante codificación abierta.

La codificación inicial de los datos fue realizada por el investigador principal y revisada por un segundo investigador con experiencia en investigación cualitativa. Esta revisión permitió ajustar códigos y mejorar la coherencia y la rigurosidad del análisis. Además, para garantizar el rigor metodológico, se aplicaron los criterios de calidad propuesto por Salgado Lévano ^27^. La credibilidad se aseguró mediante la validación de los hallazgos con las participantes. Se describe el contexto del estudio para facilitar la transferibilidad de los resultados. La dependibilidad se logró a través de una inmersión consistente en el campo y la confirmabilidad mediante la revisión de los resultados por los investigadores expertos en la metodología y el referencial teórico empleado en este estudio.

Para el análisis de los datos se empleó el software ATLAS.ti versión 8.0. La codificación se realizó de manera inductiva, construyendo un libro de códigos inicial a partir de las primeras cinco entrevistas que generó 50 códigos. Posteriormente, se efectuó un proceso de validación con las siguientes cinco entrevistas, en el cual, con el acompañamiento de un experto en el marco teórico, se fusionaron códigos y se ajustaron definiciones para lograr mayor coherencia. El resultado final fue un libro de códigos consolidado con 36 códigos, organizados en 10 unidades de registro y tres temas para el análisis final.

### Aspectos éticos

El proyecto fue aprobado por el Comité de Ética e Investigación de la Facultad de Medicina de la Universidad Nacional Mayor de San Marcos y el Comité de ética de la Dirección de Redes de Salud de Lima Sur. Asimismo, fue registrado en PRISA (Registro de Proyectos de Investigación en Salud). Al inicio, se brindó a las cuidadoras información detallada sobre los objetivos y procedimientos de la investigación. Antes de cada entrevista, se obtuvo el consentimiento informado verbal y se solicitó autorización para grabar la sesión. El anonimato de las participantes se garantizó mediante la asignación de seudónimos inspirados en nombres de la mitología griega.

## RESULTADOS

Participaron 15 cuidadoras, cuyas características se presentan en la tabla 1.

**Tabla 1:** Características de las cuidadoras entrevistadas.

Seudónimo	Fue diagnosticada con TB	Edad	Estado civil	Grado de instrucción	Parentesco
Zeus	Sí	24	Madre soltera	Secundaria completa	Mamá
Hera	No	32	Casada	Secundaria completa	Mamá
Poseidón	Sí	35	Conviviente	Secundaria completa	Mamá
Afrodita	Sí	36	Casada	Secundaria completa	Mamá
Ares	Sí	25	Casada	Secundaria completa	Mamá
Atenea	No	56	Conviviente	Primaria completa	Abuela
Hermes	Sí	48	Casada	Secundaria completa	Mamá
Apolo	Sí	24	Casada	Secundaria completa	Mamá
Artemisa	Sí	45	Casada	Secundaria completa	Mamá
Gea	No	34	Casada	Secundaria incompleta	Mamá
Hefesto	Sí	43	Casada	Secundaria completa	Mamá
Cronos	No	35	Casada	Secundaria completa	Mamá
Deméter	Sí	41	Casada	Secundaria incompleta	Mamá
Hestia	Sí	27	Conviviente	Secundaria completa	Madrastra
Dionicio	No	40	Casada	Secundaria incompleta	Mamá

### Temas que reflejan el imaginario social en la cotidianidad de la cuidadora

En análisis temático de las declaraciones permitió identificar 449 unidades de registro, que conforman tres temas: a) identificando potencias y límites del afrontamiento a la TB, b) el impacto del imaginario social en las practicas del cuidado sensible durante el tratamiento preventivo, y c) interpretando la construcción social de la tuberculosis y sus prácticas preventivas.

### a) Identificando potencias y límites del afrontamiento a la TB

Conformado por dos subtemas. Las declaraciones de los participantes que ilustran cada uno de estos subtemas se detallan en la tabla 2.

**Tabla 2:** Subtemas y declaraciones reportadas como potencias y límites para afrontar la tuberculosis.

Subtema	Declaraciones
Potencias	*- Ya estaría muerta, hasta en el hospital; pero, gracias a Dios, por estos medicamentos, yo sigo continuando mi vida y también con mis hijos* (Poseidón). *- No sé si presentará alguna reacción; espero que no, que esté tranquilo solamente. Hay que agradecer a Dios, que todo va a estar bien con nuestros hijos* (Hera). *- Gracias a Dios que en estas épocas ya hay tratamientos, no es como antes cuando no había tratamiento, las personas que se enfermaron, fallecieron, la mayoría. He luchado, gracias a Dios, salí de esto y ya, pues, ahí estamos* (Hermes). *- Me dijeron sobre la enfermedad, de la fiebre por más de 15 días; pero ya no me acuerdo y no quiero pensar en esa enfermedad, porque es horrible* (Hera). *- Pero, después, poco a poco, ya comprendí. A veces, pasan cosas en la vida que tiene que superarse nomás. Yo creo que hay que aprender a sobrevivir con todo lo que pasa y tengo que superar* (Gea). *- Al principio, no podía ir a mi casa. Tampoco mi familia sabía de lo que yo tenía, sentía un poco de vergüenza, me sentía sola. Pasando los meses, como me dijo la enfermera*: *«Después de los 2 meses ya uno no contagia, igual fui con mi mascarilla, hablé con mi familia y me ayudaron, me apoyaron»* (Ares). *- Mi esposo me brinda bastante apoyo emocional y moral, me apoya bastante, me lleva a mis controles, va a recoger mis pastillas, dentro de lo que puede* (Atenea). *- Me trataron bien, me explicaron que eso se contagiaba por estar estornudando o tosiendo, que tengamos cuidado. A mí el personal de salud me ayudó bien, yo sí entendí, bueno al principio no* (Ares). *- Acá en la posta* (establecimiento de salud) *excelente, me dijeron: «Tienen que tomar estas pastillas, que es para prevención», por lo mismo que yo estaba pasando, era protección para ellos. Las enfermeras son de maravilla, unas buenas personas; al toque, te atienden; con su ayuda, accedí al SIS* (Seguro Integral de Salud) (Deméter).
Límites	*- Fue de la nada…a mí la enfermedad me chocó…, yo dejé de comer. Es un gran dolor y peor estar mal y que tu familia te vea así, es más triste. Me sentí mal porque, no podía hacer nada, no podía trabajar ni atender a mis hijas, estaba mal, me he alegado un poco de ellas…tenía miedo porque decía que con eso me moriré* (Zeus). *- Pensaba que iba a morir, porque sentía mis pulmones raros, ya no podía ni respirar muy bien, como ahora que hago un respiro profundo…pensaba que por más que tomara la pastilla me iba a morir, tengo miedo a decaer y esta vez sí morirme y dejar a mis hijitos que son chiquitos* (Poseidón). *- Primero comienzos, como que no podía trabajar, tenía que estar en casa, nomás no podía hacer fuerza…paraba tosiendo; pero, después de tres meses, ya empecé hacer mis cosas, normal* (Zeus). *- Cuando te enfermas evitas varias cosas, no estas con tu familia porque te discriminan…como que me marginaba algo así. Cuando yo le tocaba me decía: «No me toques, que me puedes contagiar»* (Zeus). *- Recibí un mal trato en el mismo centro médico, un trato discriminatorio de la señorita* (personal de limpieza), *lo que me afectó psicológicamente, porque estaba muy sensible* (Dionisio). *- Me mandaron a mi casa, total que yo estuve así más de una semana, me sentí mal y como vino el COVID-19, ya no que quise exponer…, me fui a un particular* (Artemisa). *- Por la enfermedad del virus, no hay atención en los hospitales, no hay a dónde ir. Todo está colapsado, eso me altera y preocupa* (Atenea).

En cuanto al subtema potencias, las estrategias de afrontamiento de las cuidadoras, como la esperanza y el agradecimiento a Dios, se interpretan como rituales que les otorgan significado a su experiencia. La comunicación del diagnóstico de TB a la familia, tras superar el miedo, funciona como un rito de iniciación que las reintegra en una tribu afectiva, brindándoles un sentido de pertenencia y apoyo. El personal de salud y las redes de apoyo actúan como catalizadores para la formación de comunidades electivas, que refuerzan el verdadero lazo social (socialidad) y la adherencia al tratamiento preventivo.

Respecto al subtema límites, el temor al contagio, el miedo a la muerte, malestar y preocupación revelan representaciones sociales de fragilidad y estigma. La vivencia de la disminución de la fuerza física, que limitó la realización de sus actividades cotidianas, subraya cómo los síntomas de la TB no solo alteran la función biológica, sino que también merman la energía vital percibida, reconfigurando la vida diaria y el sentido de autonomía de los individuos. El trato discriminatorio de familiares y del personal de limpieza fue visto como una exclusión de la tribu social, rompiendo lazos de apoyo. Finalmente, las restricciones por la pandemia fragmentaron los rituales institucionales y las redes de apoyo, dejando a las cuidadoras en una situación de mayor vulnerabilidad.

### b) El impacto del imaginario social en las prácticas del cuidado sensible durante el tratamiento preventivo

Emergieron tres subtemas. Las declaraciones de los participantes que ilustran cada uno de estos subtemas se detallan en la tabla 3.

**Tabla 3:** Subtemas y declaraciones reportadas sobre el impacto del imaginario social del cuidado sensible durante el tratamiento preventivo

Subtema	Declaraciones
Estar y cumplir juntos el tratamiento	*- Mi hijo tenía que tomar su pastilla después del desayuno, todas las mañanas a las 8 a.m. ….. Debemos apoyarles, no dejarlos solos, siempre estar con ellos, … Hacerle su comida, estar siempre a su lado; es lo más importante, porque si no tampoco se va a mejorar* (Gea). *- Ahora, como ya estoy acá, paro más pendiente de él* (hijo)*, con su alimentación para que tome un buen desayuno y al tomar su pastilla, no le caiga pesado o no le afecte …Normalmente, le doy las pastillas en la mañana, después del desayuno, mayormente con agua …* (Atenea). *- A los niños se les debe inculcar sobre la importancia de tomar las medicinas, por ejemplo, a mi hijo (14 años) le hago ver los videos de internet acerca de la enfermedad o de lo que puede pasar si deja de tomar las pastillas. Entonces, le digo: «Si cumplimos esta fase y lo pasamos, listo y feliz. Pero, si no, al año, nos van a tener que inyectar»* (Afrodita). *- … Ellas tomaban después de desayunar, les daba en juguito, como les gusta el jugo de fresa, lo mezclaba con eso y les hacía además, le metía un poquito de ‘miel’ para que no se sienta …* (Zeus). *- Desde que me dijeron que mi hijo debía de tomar las pastillas, yo le di; aunque su papá me decía: «¿por qué lo vas a dar?», y cosas así. Sin embargo, yo agarré valor y le comencé a dar las pastillas, hasta que terminé su tratamiento* (Hera). *- A veces, me olvidaba darles las pastillas por las tareas que tenían ellos, por las clases. También porque tenía que irme al mercado, otras veces por tantas preocupaciones que tengo personalmente, fue por eso también* (Poseidón).
Interactuando con el niño	*- Cuando empezó a tomar (isoniazida), le dije: «Hija, esto es para prevenir, para que no estés igual que yo, tienes que tomar», ella agarraba, me decía: «Mamá, dame mi medicina»* (Zeus). *- No les llegué a decir a mis hijos, porque no quería que se enteraran de que yo tenía TB y no me preguntan mucho tampoco. Solamente les dije que eran unas pastillitas que tenían que tomar para que no les dé anemia y no se enfermen, ni nada de eso* (Ares). *- Él* (hijo) *estuvo tomando* (tratamiento preventivo), *pero, así como todo joven como que un poco no quería tomar, de por sí hay que estar detrás de él* (hijo) *para que tome porque decía que estaba bien* (Atenea). *- …ella dejó tomar y lo dejó faltando un mes, porque creo que dijo que no quería tomar … Ella misma me dijo que no, como que le hacía sentir mal, que bajó de peso, prácticamente perdió el apetito* (Deméter).
Actitudes favorables al tratamiento	*- Cuando me dijeron que mis niñas iban a iniciar tratamiento con esas pastillas, yo pensaba que en realidad tendría esa enfermedad, que yo tenía, eso me puse a pensar* (Zeus). *- Tenía que tomar estas pastillas para que no se enferme de eso más adelante, porque ha estado en contacto con mi hermano por más de un mes, sin saber que tenía esa enfermedad* (TB) (Hera). *- Me dijeron que no estaban contagiadas; pero que tenían la enfermedad, algo así. «… no me acuerdo muy bien» Solo me dieron como prevención, las pastillas* (Ares). *- El motivo que tuve para que mis hijas cumplieran con el tratamiento era porque yo no me quería sentir mal, … no quería exponerlas, tal vez no por mi salud, … porque era una responsabilidad por parte mía* (Ares). *- … A mí también me afectaba mucho, el primer mes casi me deprimo porque yo decía que por mi culpa mi hija estaba así con todos esos síntomas.* (Dionisio).

En cuanto al subtema *Estar y cumplir juntos el tratamiento*, la administración de las pastillas no es solo un acto de cuidado, sino un ritual cotidiano de protección que esta influenciado por sus emociones, creencias y vínculos sociales. Las cuidadoras, al aplicar estrategias y cumplir con la tarea, refuerzan un sentido de pertenencia en la «tribu familiar», lo que consolida su compromiso con la prevención.

En el subtema *Interactuando con el niño*, las cuidadoras emplearon diversas estrategias de comunicación. Una de ellas fue una narrativa de prevención, que consolidó prácticas de cuidado y facilitó la adhesión. No obstante, esta aproximación no fue uniforme. Algunas cuidadoras optaron por ocultar la enfermedad, mientras que otras tuvieron que gestionar directamente la resistencia de los niños al tratamiento.

Respecto al subtema *Actitudes favorables al tratamiento*, las percepciones sobre el tratamiento y el sentimiento de culpa como motor de la responsabilidad actúan como mecanismos afectivos que solidifican la identidad de la cuidadora, impulsando la adherencia al ritual y el compromiso de la tribu familiar.

### c) Interpretando la construcción social de la tuberculosis y sus prácticas preventivas

Conformado por tres subtemas. Las declaraciones de los participantes que ilustran cada uno de estos subtemas se detallan en la tabla 4.

**Tabla 4:** Subtemas y declaraciones reportadas sobre el tema interpretando la construcción social de la tuberculosis y sus prácticas preventivas

Subtemas	Declaraciones
Imaginario de las prácticas para prevenir la TB en su hogar	*- También tenía que separar mis platos, mis cubiertos, todo eso para no tener mucho contacto con mis hijas, … la señorita (enfermera) también me dijo: «Ya no puedes contagiar», porque estoy tomando los medicamentos, ya no puedo contagiar, pero con mis cubiertos todo aparte* (Zeus). *- Le puse una marquita a mi taza, mi cuchara, a todo. Ya todos sabían y no agarraban mi taza* (Hestia). *- Yo trabajé en una guardería, en la cocina, siempre recibimos charlas de nutrición. Entonces, a base de eso, yo he tratado de alimentar a mis hijos de una u otra manera con bastante menestra, huevo, leche y verduras. Aunque de vez en cuando les compraba sus vitaminas como refuerzo. Además, me he puesto a hacer más limpieza. Quizás exageré en lo que sería la limpieza* (Hermes). *- La tuberculosis se previene con bastante alimento, comiendo. Eso me dijo (personal de salud). Le di carne de cuy porque yo lo comía cuando tuve anemia más antes, cuando di a luz* (Gea). *- Me descuidé en el tema alimenticio, comía en la calle. Ahí es que sentí los síntomas. Empecé a toser con sangre. También, quizá cuando bajé las defensas* (Zeus). *- La mayoría lo tiene por falta de higiene, precaución o bajas defensas. Cuando baja las defensas, esa enfermedad evoluciona y te ataca. Bueno, entonces, hay que alimentarse sanamente* (Deméter). *- Tenía que mandarle a la casa de mi suegra para que pueda estar bien, para no contagiarlas… y a veces cuando vienen mis hijas, pues como son chiquitas, quieren abrazarme y yo no puedo. Trato un poquito de tomar distancia con ellas por la enfermedad* (Zeus). *- A mi hijo mayor, le dije que tengo una herida en el pecho, en mi pulmón y que tengo que estar con las mascarillas para no poder toser y contagiarles a ellos. No he tenido tos, pero siempre ando con la mascarilla y trato de lavarme mis manos antes de tocar las cosas de mis hijos* (Apolo). *- Hoy en día, que estamos atravesando la enfermedad de la COVID-19, no salimos, paramos acá en el cuarto. Siempre se lava las manitos con jabón líquido* (Hera). *- La enfermera me dijo: «Deberías vivir en un lugar ventilado y dormir aparte». Pero la situación está difícil, nadie alquila barato y no hay dinero. Por eso, hemos roto un pedacito del triplay (pared) para hacer una ventana chiquita, para que pueda entrar el aire* (Poseidón). *- También, abro mis ventanas para que entre el solcito* (Hera).
Apoyo en el control de contactos	*- Cuando su hermano inició el tratamiento (anti-TB), evaluaron a mi hijo y me dijeron que estaba en riesgo. Entonces, le hicieron un descarte, le sacaron placas del pulmón; a mí solo me sacaron de esputo, pero el resultado salió negativo* (Hera). *- A mi hijita, le mandaron hacer una prueba. Entonces, llevé a mi hija para que le hagan la prueba en el antebrazo (prueba de tuberculina).y a todos los que estaban alrededor de mi familia. Gracias a Dios, todos salieron negativos* (Artemisa). *- Es que mi hijito estaba con tos fuerte cuando yo inicié el tratamiento para la TB. Pero, parecía como un bronquio fuerte, le tuvieron que sacar una placa y el neumólogo, quien me dijo que tenía bronquios mal curado* (Apolo). *- Le sacaron plaquitas de los pulmones, al comienzo. Después, al intermedio y al final…. Después, fue el examen de la saliva* (baciloscopia)*, que también me mandaron; así constantemente. Último también, no me recuerdo, pero sí era el control que le hacían* (Hermes).
Imágenes de la TB	*- Bueno, sé que prácticamente es una enfermedad común, yo ya he tenido familiares que han pasado por esto, 2 hermanos, 2 cuñados. Entonces sé que es curable, esta enfermedad es contagiosa si no hay prevención o cuidado…* (Deméter). *- Solamente sabía que la TB era una enfermedad contagiosa y que afecta a los pulmones. Yo no sabía mucho a nadie de mi familia le había dado. Por eso, se me hizo un poco difícil asumir la enfermedad* (Ares). *- …, mis familiares que han muerto supuestamente, fue porque dejaron el tratamiento debido a que le hacía daño, avanzó la enfermedad y se murieron … si a mí no me dan pastillas, ya estaría muerta, hasta el hospital he llegado* (Poseidón). *- Es complicado, es una enfermedad muy seria, porque pueden pasar cosas mayores, lo que me han explicado a mí .. que se cura, pero siempre llevando el tratamiento al pie de la letra. Imagine, pueden pasar cosas mayores como es la muerte* (Atenea). *- Me dicen que si tenía de tres niveles, no recuerdo muy bien. O sea, lo mío estaba bien fuerte y me preguntaba qué había pasado porque hacía tiempo que no me enfermaba de la tos, de la fiebre.; pero esta vez era diferente, muy fuerte ha sido*” (Poseidón). *- En el caso de mi hermano, estaba en gravedad, porque estaba botando sangre y no tenía tos, ni yo tampoco. Simplemente que un día se fue a trabajar, se tomó una gaseosa helada, ahí le empezó a doler la espalda luego a botar sangre y fiebre* (Deméter). *- Porque el mío creo que es de menor grado, creo que hay tuberculosis de diferentes grados* (Hestia). *- Me informaron que era una vacuna y que con eso íbamos a saber si estábamos contagiados o no. Después de que le pusieron, a los cuatro días, vine con mis hijos, me dijeron que estábamos contagiados* (Poseidón). *- Me mandaron a sacar otro esputo, también a sacar placas … y pase con el doctor. Me dijeron: «Efectivamente, tiene la tuberculosis y debe de seguir el tratamiento durante 6 meses»* (Artemisa). *- Cuando me llama la enfermera, me dice que tengo la TB número tres, no recuerdo bien, pero me dijeron que eso significaba que puedes contagiar a tus hijos* (Poseidón).

En el primer subtema, imaginario de las prácticas para prevenir *la TB en su hogar* se manifiesta en rituales de higiene y protección. La separación y marcación de utensilios, junto con la limpieza «excesiva», constituyen rituales de purificación que buscan controlar el miedo al contagio y establecer fronteras simbólicas en la «tribu familiar». Las prácticas de distanciamiento durante la pandemia por COVID-19 se convirtieron en ritos de seguridad colectiva. La promoción de la ventilación, incluso en precariedad, demuestra la adaptación de esta percepción a su realidad.

El segundo subtema muestra cómo las practicas simbólicas de cuidado se extiende más allá del núcleo familiar inmediato. El apoyo en el control de contactos, al acompañar a familiares en los exámenes de descarte, se interpreta como un ritual de solidaridad y cohesión tribal. Al considerarlos en riesgo, se refuerza la idea de una comunidad de destino unida por una amenaza compartida. Favorecer el acceso a los servicios de salud consolida esta red de apoyo como un mecanismo de protección colectivo, reforzando los lazos afectivos de la «tribu».

En el tercer subtema, las imágenes de la TB se construyen a partir de conocimientos y percepciones. La descripción de la TB como una enfermedad contagiosa revela representaciones sociales basados en el miedo y la prevención. La creencia de que la falta de tratamiento puede causar la muerte es una narrativa de riesgo que justifica y da sentido a las prácticas de cuidado. Las cuidadoras asignan niveles de gravedad a la enfermedad según los síntomas, lo que muestra que este imaginario no es estático, sino que se reconfigura con la experiencia.

## DISCUSIÓN

Este estudio, orientado a interpretar el imaginario social de un grupo de cuidadoras de niños en TPTB, reveló que al afrontar la TB, identifican potencias y límites en su día a día. Luego de asumir su rol, ofrecieron un cuidado sensible al niño, atravesado por emociones, vivencias, experiencias, creencias y vínculos sociales, lo que se manifestó en rituales de protección y en la construcción de una narrativa de prevención. Simultáneamente, construyeron y expresaron un significado particular de la TB y sus medidas preventivas, adaptando sus prácticas de higiene a su contexto socioeconómico y el conocimiento ordinario. Estos actos simbólicos y significados fueron moldeados por sus miedos, incertidumbres y creencias durante sus vivencias con la familia y el personal de salud, así como por un profundo sentido de responsabilidad y solidaridad.

En el entorno familiar, al comunicar su diagnóstico, las cuidadoras con TB recibieron apoyo familiar (potencia), aunque algunas enfrentaron situaciones de maltrato y discriminación (límite). De manera similar, investigaciones cualitativas realizadas en Estambul ^28^ y Colombia ^29^^)^ destacan que el apoyo social de la familia y amigos representa el principal soporte para afrontar la TB. Asimismo, otro estudio reportó que los afroamericanos en Estados Unidos fueron discriminados por sus familiares ^30^; además, en Perú se ha documentado que el acompañamiento de la familia y del personal de enfermería permitió a los pacientes superar la discriminación y el estigma social ^31^. Estos apoyos evidencian el «entre-ensemble» pues se da en el «estar juntos» que convoca a una dinámica de solidaridad y «sentir juntos», que alude a la ética de la estética ^32^.

En cuanto al personal de salud de los servicios de TB, las cuidadoras también vivieron experiencias positivas, como la orientación y el apoyo (potencia); sin embargo, reportaron situaciones de discriminación (límite), principalmente por parte del personal de limpieza. La discriminación en TB puede estar relacionada con la asociación de la enfermedad a estigmas sociales y culturales ^33^. Las nociones de potencias y límites también emergieron en estudios cualitativos que analizaron la cotidianidad de los estudiantes de enfermería durante su formación ^22^, así como en la atención en salud a personas sordas ^23^.

El cuidado sensible que brindan las cuidadoras se articula en una dualidad entre la razón y el sentimiento, lo cual les permite brindar un cuidado holístico y centrado en la persona, que trasciende las tareas y se enfoca en el bienestar emocional y físico de quien recibe el cuidado. Esto evidencia que la forma en que se ofrece el cuidado puede influir en la experiencia de quien lo recibe ^22^. Además, se incorporan aspectos como la empatía y la creatividad para atender las necesidades psicoemocionales, el sufrimiento y la ansiedad que genera la enfermedad ^34^. La evidencia disponible demuestra que el cuidado sensible materno tiene múltiples beneficios importantes como promover la seguridad en el apego ^35^ y el desarrollo cerebral infantil ^36^.

Al otorgar significado a la tuberculosis en su vida cotidiana, las participantes demostraron un compromiso y una resistencia multifacética. Un primer hallazgo mostró la «ética de la estética», presente en la sociabilidad, que permite redescubrir la gracia de estar y sentir juntos, de conectar con los demás, para vivir sanos y a diario ^37^, donde la empatía y la conexión emocional con el niño fueron clave para la adherencia al tratamiento. Por otra parte, el uso de dispositivos electrónicos e Internet para negociar el cumplimiento del tratamiento alude a que, en la posmodernidad, existe una sinergia entre lo «arcaico y el desarrollo tecnológico^» (^^32^. Este hallazgo se complejiza por el hecho de que, en ocasiones, el compromiso era una «máscara» social que se desvanecía en la privacidad del hogar, debido a presiones socioeconómicas y a la multiplicidad de roles que las cuidadoras asumen, lo que condujo al descuido en la administración de la medicación. Este contraste central revela la teatralidad de la vida cotidiana y la complejidad de las experiencias de las cuidadoras. Se alude aquí a la alegoría del «juego de las máscaras», donde las personas tienden a adoptar diferentes roles y máscaras sociales en función del contexto en que se encuentran. En ese sentido, la «máscara» representa la dimensión dramática de la entretejida experiencia y la teatralidad de la vida cotidiana ^24^.

El análisis del imaginario social en la cotidianidad de las cuidadoras reveló una integración de la razón y el sentimiento. La «crítica al dualismo» ^22^ se refleja en el equilibrio entre la racionalidad para afrontar el diagnóstico de TB y el apoyo en la espiritualidad y la fe para encontrar fortaleza emocional. Esta dualidad muestra que la gestión de las emociones trasciende lo físico, ya que involucra también lo emocional y espiritual ^21^. Por otro lado, el presupuesto del «formismo», subraya la prioridad que las cuidadoras otorgan a la armonía en sus prácticas de cuidado, buscando una estética de coherencia en sus acciones. El «pensamiento libertario» emergió en la capacidad de las cuidadoras para generar estrategias innovadoras, demostrando que lo cotidiano es un espacio de resistencia a la imposición externa. Además, la sensibilidad relativista ^21^^),^ permitió entender que el significado de la TB no es fijo, sino que se basa en las percepciones individuales y en el conocimiento ordinario ^13^ que las cuidadoras construyeron a partir de la observación de los síntomas y de la interacción con el personal de salud. Este conocimiento no se opone al científico, sino que lo complementa.

Este estudio tiene implicancias prácticas para la prevención de la TB, considerando que los conocimientos y percepciones están asociados con el cumplimiento de la TPTB ^9^. Estos hallazgos sugieren que el personal de salud debe tener en cuenta estos aspectos al implementar intervenciones educativas o al brindar consejería individualizada. Asimismo, para lograr un abordaje integral del fenómeno de adherencia o cumplimiento del tratamiento, se necesita develar las diferentes «máscaras» que se coloca la cuidadora mediante acompañamiento, trato cordial y consideración de sus sentimientos, temores y miedos. Desde el punto de vista metodológico, esta investigación es pionera en abordar la prevención de la TB desde la sociología del cotidiano, describiendo potencias y límites en el afrontamiento de la enfermedad; estas nociones pueden ser identificadas como dimensiones internas y externas de la persona, en su relación con la familia, el personal de salud y su entorno.

El estudio no estuvo exento de limitaciones. En primer lugar, por tratarse de un estudio cualitativo, sus hallazgos no pueden ser generalizables a toda la población de cuidadores de niños en TPTB del Perú. En segundo lugar, debido a las restricciones impuestas por la pandemia de COVID-19, fue necesario realizar entrevistas a profundidad virtualizadas, mediante el uso de las plataformas digitales; las cuales, en algunas oportunidades, presentaron interrupciones por problemas técnicos. En tercer lugar, respecto a la comunicación, se perdió información no verbal crucial, especialmente durante las llamadas telefónicas y dificultades para establecer el *rapport* adecuado con los participantes y afectó, en cierta medida, la riqueza de los datos recolectados en comparación con entrevistas presenciales. Sin embargo, se contó con una guía de entrevista para asegurar la estandarización del proceso de recolección de datos y el investigador encargado contaba con experiencia en investigación cualitativa. En cuarto lugar, la ausencia de triangulación metodológica para garantizar la fiabilidad de los datos constituye otra limitación importante. Como quinta limitación, el contexto de la pandemia pudo haber influido el comportamiento de las cuidadoras debido al aumento de la convivencia con los niños. Por lo tanto, estos hallazgos deben interpretarse considerando las condiciones excepcionales de ese período, las cuales ya no están vigentes. Finalmente, el reducido número de participantes considerado hasta alcanzar el punto de saturación podría no reflejar completamente la diversidad de experiencias o perspectivas sobre el tema estudiado; no obstante, esta muestra fue diversa, ya que incluyó personas diagnosticadas con TB y contactos sin esta enfermedad de cuatro establecimientos de salud.

En conclusión, este estudio revela que el imaginario social en la cotidianidad de las cuidadoras de niños en TPTB es un constructo dinámico y complejo. Se evidenciaron límites y potencias durante el afrontamiento de la TB en su vida diaria. El cuidado sensible que brindaron estuvo influenciado por sus emociones, vivencias, experiencias, creencias y vínculos sociales. Asimismo, el conocimiento y la comprensión de la enfermedad no son puramente racionales, sino que estaban impregnados por el imaginario colectivo, en el cual los miedos, la incertidumbre y las creencias funcionan como un filtro a través de los cuales se interpretó y resignificó la experiencia. Estos hallazgos sugieren que, en la prevención de la TB, es crucial adoptar un enfoque más empático, espiritual y personalizado que valore la creatividad, la empatía y la resiliencia de las cuidadoras, considerando sus experiencias, vivencias y creencias. Finalmente, para mejorar la adherencia a la TPTB, es esencial que el personal de salud sea capacitado en habilidades de escucha activa y empatía para reconocer las «máscaras sociales» que ocultan los temores y problemas de las cuidadoras. En esencia, la prevención de la TB debe dejar de ser una imposición para convertirse en un proceso de acompañamiento que valore la inclusión y la sensibilidad; integrando el apoyo comunitario y la espiritualidad.
